# Bulk Plasmon Polariton Modes in Hyperbolic Metamaterials for Giant Enhancement of the Transverse Magneto-Optical Kerr Effect

**DOI:** 10.3390/molecules27165312

**Published:** 2022-08-20

**Authors:** Brayan Fernando Díaz-Valencia, Edwin Moncada-Villa, Faustino Reyes Gómez, Nelson Porras-Montenegro, Jorge Ricardo Mejía-Salazar

**Affiliations:** 1 Departamento de Física, Universidad del Valle, A.A., Cali 25360, Colombia; 2 Escuela de Física, Universidad Pedagógica y Tecnológica de Colombia, Avenida Central del Norte, Tunja 39115, Colombia; 3 Instituto de Física de São Carlos, Universidade de São Paulo, P.O. Box 369, São Carlos 13566-590, Brazil; 4 Instituto Nacional de Telecomunicações (Inatel), Santa Rita do Sapucaí 37540-000, Brazil

**Keywords:** magneto-optical materials, TMOKE, magnetoplasmonics

## Abstract

We demonstrate a concept for the giant enhancement of the transverse magneto-optical Kerr effect (TMOKE) using bulk plasmon polariton (BPP) modes in non-magnetic multilayer hyperbolic metamaterials (HMMs). Since the BPP modes are excited through the attenuated total reflection (ATR) mechanism, using a Si-based prism-coupler, we considered a single dielectric magneto-optical (MO) spacer between the prism and the HMM. The working wavelength was estimated, using the effective medium approach for a semi-infinite dielectric-plasmonic multilayer, considering the region where the system exhibits type II HMM dispersion relations. Analytical results, by means of the scattering matrix method (SMM), were used to explain the physical principle behind our concept. Numerical results for giant TMOKE values (close to their maximum theoretical values, ±1) were obtained using the finite element method (FEM), applying the commercial software COMSOL Multiphysics. Our proposal comprises a simple and experimentally feasible structure that enables the study of MO phenomena in HMMs, which may find application in future nanostructured magnetoplasmonic metamaterials for active nanophotonic devices.

## 1. Introduction

During the last decade, diverse and important results from theoretical and experimental research on the implementation of magneto-optical (MO) materials in photonic systems have been obtained. Of particular interest are applications of the transverse magneto-optical Kerr effect (TMOKE) in areas such as optical isolators [1], optical filtering devices [2], extraordinary optical transmission [3], data storage devices [4,5], magnetic field sensors [6], and high-sensitivity optical biosensors [7,8], among others. TMOKE measures the relative change in the reflectance ($R_{pp}$) of *p*-polarized light impinging on the surface of an MO material, when the magnetization M (placed transverse to the plane of incidence) is reversed [9]

(1) $$\mathrm{TMOKE}=\frac{R_{pp}\left(+M\right)-R_{pp}\left(-M\right)}{R_{pp}\left(+M\right)+R_{pp}\left(-M\right)}.$$

Although this MO effect is of the order of $10^{-3}$ in conventional ferromagnetic surfaces [9], hindering applications, researchers have demonstrated enhancements of up to three orders of magnitude when using a nanostructured metallic surface, where the surface plasmon resonances (SPRs) are excited [10,11,12,13]. An important application of these nanostructures is magnetoplasmonic biosensing [14,15,16,17], which merges the sharp resonances of TMOKE with the high sensitivity of SPRs to small changes in the surrounding dielectric environment for improved sensing resolutions. The latter mechanism exploits highly enhanced and localized optical fields (at the metal surface) to enhance the MO activity of an adjacent ferromagnetic material [10].

On the other hand, MO effects in hyperbolic metamaterials (HMMs) have attracted research attention over recent years [18,19,20,21]. From a fundamental point of view, MO-HMMs allow study of the interplay of plasmonic, magnetic, and dielectric properties in a single highly anisotropic material [19,20,21], whereas, from an applications perspective, the unique optical properties of MO-HMMs enable the design and development of highly integrated (with enhanced resolution) (bio)sensing devices [22,23]. HMMs are classified according to the signs of their tangential ($\varepsilon_{\|}$) and vertical ($\varepsilon_{⊥}$) permittivity components as type I, for $\varepsilon_{\|}>0$ and $\varepsilon_{⊥}<0$, and type II, for $\varepsilon_{\|}<0$ and $\varepsilon_{⊥}>0$ [24]. Among the existing fabrication methods for HMMs, pulsed laser deposition [25], thermal and electron-beam evaporation [26], and nanolithography techniques [27] can be mentioned. The uniaxial feature of HMMs is reached through the use of two-dimensional arrangements of plasmonic nanorods [25] (disposed in a dielectric host) or by alternating dielectric and plasmonic slabs in multilayer structures [26,27,28]. In the case of MO-HMMs built by nanorod arrays, the MO activity comes from the inclusion of ferromagnetic-metallic shells (e.g., of Ni) around the nanorods [18,19,20,21] or by using a magnetoplasmonic substrate [22]. In contrast, multilayer MO-HMMs are composed of alternating plasmonic and dielectric MO slabs [23], which have recently been used for the giant enhancement of TMOKE through the collective excitation of SPRs at each dielectric MO/metal interface in the multilayer system [23,29]. These collective excitations of SPRs lead to electromagnetic field localization within the structure and are, therefore, commonly known as multilayer plasmons, volume plasmon polaritons (VPPs), and bulk plasmon polariton (BPP) modes [26,28,30]. We will use the latter terminology in this paper. Despite the high-quality TMOKE resonances demonstrated with multilayer MO-HMMs [23], interest in this physical mechanism for enhancing MO activity (and its applications) is still limited. In particular, the complex combinations of growth-steps for precise deposition of ferromagnetic materials within each unit cell of the structure could be challenging.

Here, we demonstrate numerically that bulk plasmon polariton (BPP) modes from a non-magnetic HMM can be used to enhance the MO activity of a single adjacent dielectric MO slab. Our proposal comprises a single Si-compatible MO-garnet slab (cerium-substituted yttrium iron garnet (Ce:YIG) [31,32,33] in this work), on a Si substrate (used as the incident medium), covered by alternate layers of Au and SiO${}_{2}$. The BPP modes in the plasmonic/dielectric multilayer HMM structure are excited through an attenuated total reflection (ATR) mechanism, using the refractive index contrast at the Si-(Ce-YIG) interface. Importantly, we used rigorous analytical calculations, with the scattering matrix method (SMM), to demonstrate the physical origin of the enhanced MO activity in the proposed platform. Quality factors (*Q*) of the order of $Q∼10^{2}$ were observed for the resonant TMOKE peaks, which could find application in future magnetophotonic-based devices.

## 2. Theoretical Framework

For SPR excitation, we consider an obliquely incident transverse-magnetic (TM) polarized electromagnetic wave of frequency $\omega =\frac{2\pi c}{\lambda }$ (λ is the wavelength of incident light and *c* is the light velocity in vacuum), with yz-plane as the incident plane i.e., $k=(0,k_{y},k_{z})$. The multilayer HMM dispersion relation [34,35]

(2) $$\frac{k_{z}^{2}}{\varepsilon_{\|}}+\frac{k_{y}^{2}}{\varepsilon_{⊥}}=\frac{\omega^{2}}{c^{2}},$$

comes from an idealization of the system as a semi-infinite stack, where the parallel ($\varepsilon_{\|}$) and perpendicular ($\varepsilon_{⊥}$) components (in relation to the plane of the structure) of the effective permittivity are calculated as

(3) $$\begin{array}{c} \varepsilon_{\|}=\rho \varepsilon_{m}+\left(1-\rho \right)\varepsilon_{d}, \end{array}$$

(4) $$\begin{array}{c} \varepsilon_{⊥}=\frac{\varepsilon_{m}\varepsilon_{d}}{\left(1-\rho \right)\varepsilon_{m}+\rho \varepsilon_{d}}, \end{array}$$

with $\rho =d_{m}{\left(d_{m}+d_{d}\right)}^{-1}$ for the filling fraction of metal inclusions; $d_{m}$ and $d_{d}$ are the thicknesses of the metallic and dielectric layers, respectively. It is worth mentioning that this last effective medium approach is only valid under the condition $\lambda \gg d_{m}+d_{d}$.

The permittivity values for Au and ${\mathrm{SiO}}_{2}$ were used from experimental results in [36,37], respectively. On the other hand, the MO slab (made of Ce-YIG material) between the substrate and the HMM is considered magnetized along the *x*-axis. Therefore, the permittivity tensor for this latter slab is written as [9,38]

(5) $${\overset{˘}{\varepsilon }}_{\mathrm{MO}}=\left(\begin{array}{ccc} \varepsilon_{\mathrm{mo}} & 0 & 0\\ 0 & \varepsilon_{\mathrm{mo}} & -img\\ 0 & img & \varepsilon_{\mathrm{mo}} \end{array}\right),$$

where *g* is the gyration value of the MO medium. Considering λ=1550 nm as the working wavelength, we use $\varepsilon_{\mathrm{mo}}=5.114+0.002i$, g=0.0087−0.0002i [33], with m=±1 to indicate the magnetization sense along the *x*-axis.

## 3. Results and Discussions

**Figure 1:**
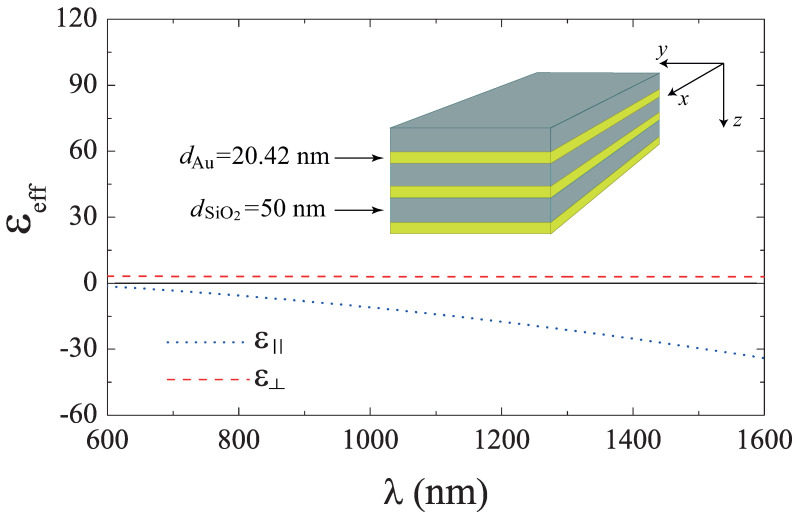
Real parts of $\varepsilon_{\|}$ and $\varepsilon_{⊥}$ for a semi-infinite multilayer built by alternate SiO${}_{2}$ and Au layers. The layer thicknesses were used as $d_{m}=20.42$ nm and $d_{d}=50$ nm for Au and SiO${}_{2}$, respectively. A schematic representation of the system under study is shown in the inset.

**Figure 2:**
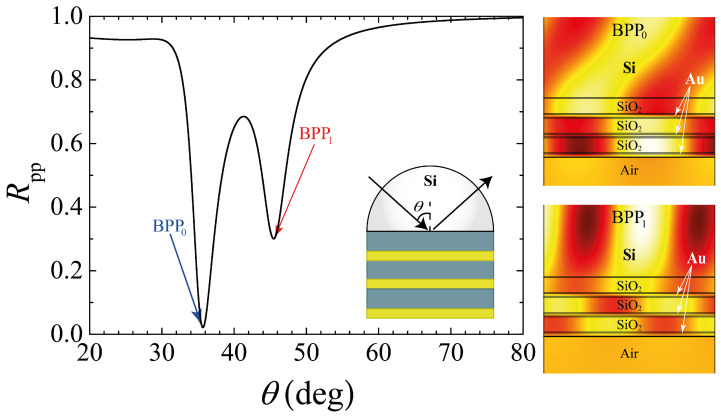
Numerical reflectance as a function of the angle of incidence. The reflectance dips labeled with BPP${}_{0}$ ($35.68^{\circ }$) and BPP${}_{1}$ ($45.48^{\circ }$) indicate the two BPP modes that are excited in the multilayer structure. The corresponding magnetic field profiles are shown in the top and bottom right side panels. The finite multilayer structure, consisting of a Si-prism and a tri-bilayer system, is depicted in the inset.

**Figure 3:**
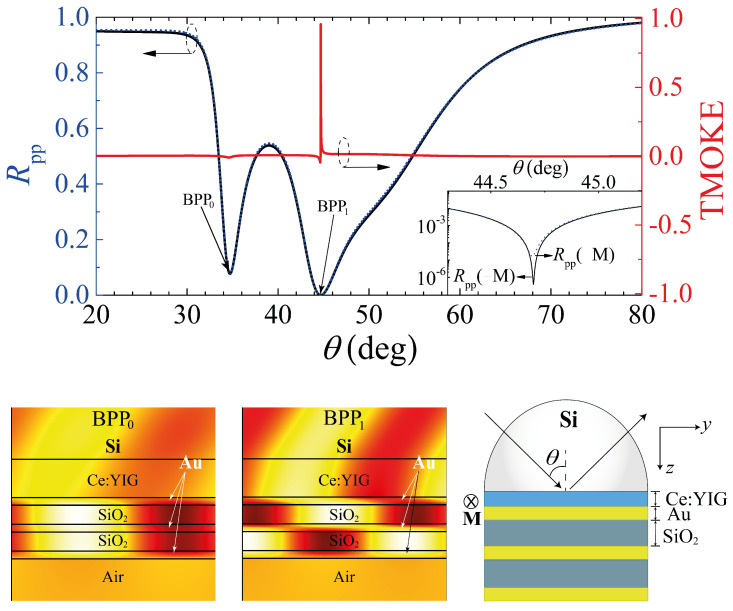
**Upper panel:** results for the reflectances $R_{\mathrm{pp}}\left(\pm M\right)$ and TMOKE are shown for comparison. The inset shows the difference between the $R_{\mathrm{pp}}\left(+M\right)$ and $R_{\mathrm{pp}}\left(-M\right)$ values around the BPP${}_{1}$ resonant angle ($44.69^{\circ }$). **Lower panel:** the magnetic field profiles $H_{x}$ (associated with the BPP${}_{0}$ and BPP${}_{1}$) and a schematic representation of the proposed MO structure, respectively. All the calculations in this figure were performed using $d_{m}=20.42$ nm and $d_{\mathrm{Ce}-\mathrm{YIG}}=100$ nm.

Let us start by discussing the effective permittivity components of an ideal semi-infinite multilayer HMM, built by alternate layers of ${\mathrm{SiO}}_{2}$ and Au (see the inset of Figure 1). The numerical results of the real parts from Equations (3) and (4) are shown in Figure 1, for $d_{m}=20.42$ nm (thickness of gold layers) and $d_{d}=50$ nm (thickness of ${\mathrm{SiO}}_{2}$ layers), calculated as a function of the incident wavelength. Since we are interested in the use of type II HMM, i.e., $\varepsilon_{⊥}>0$ and $\varepsilon_{\|}<0$, we focused our attention on the wavelength region from 600 nm to 1600 nm where, as observed, this condition is fulfilled. Then, we set the working wavelength λ=1550 nm to be far enough from the minimum λ for Type II HMM dispersion. To proceed to the study of a finite multilayer system, we first performed numerical calculations and analysis, from which we observed that multilayers with a minimum of three ${\mathrm{SiO}}_{2}$/Au bilayers can be used for the excitation of BPP modes. This last result can be seen in Figure 2, where the two reflectance minima (labeled as ${\mathrm{BPP}}_{0}$ and ${\mathrm{BPP}}_{1}$) correspond to the BPP modes for the system illustrated in the inset. Furthermore, the volumetric distribution of the magnetic field profiles, associated with these modes, can be verified from the insets on the right-hand side of this latter figure. The permittivity values for ${\mathrm{SiO}}_{2}$ and Au were used as $\varepsilon_{{\mathrm{SiO}}_{2}}=2.085$ and $\varepsilon_{\mathrm{Au}}=-108.556+10.4329i$, respectively [36]. It should be noted that the dielectric layer between the Si substrate (prism coupler) and the first Au layer (where the plasmonic excitation takes place) can be thought of as a spacer to adjust the evanescent coupling of the ATR field and the SPR phenomenon. Therefore, replacing the spacer with an MO Ce-YIG slab should enable a mechanism for active manipulation of the matching condition between the ATR and BPP fields. Using the SMM for the system schematically represented in the lower panel of Figure 3, we obtained the following equation for the corresponding eigenmodes

(6) $$i\beta_{\mathrm{Ce}-\mathrm{YIG}}\cot\left(d_{\mathrm{Ce}-\mathrm{YIG}}q_{\mathrm{Ce}-\mathrm{YIG}}\right)=\frac{F_{N}}{F_{D}},$$

with

(7) $$\begin{array}{ccc} F_{\sigma } & = & -6\beta_{\mathrm{Au}}^{3}\beta_{{\mathrm{SiO}}_{2}}^{2}\cos\left(d_{\mathrm{Au}}q_{\mathrm{Au}}\right)\cos^{2}\left(d_{{\mathrm{SiO}}_{2}}q_{{\mathrm{SiO}}_{2}}\right)\\ & & \times \sin^{2}\left(d_{\mathrm{Au}}q_{\mathrm{Au}}\right)G_{\sigma }+2i\beta_{\mathrm{Au}}^{2}\beta_{{\mathrm{SiO}}_{2}}^{2}\cos^{2}\left(d_{{\mathrm{SiO}}_{2}}q_{{\mathrm{SiO}}_{2}}\right)\\ & & \times \sin^{3}\left(d_{\mathrm{Au}}q_{\mathrm{Au}}\right)H_{\sigma }+\sin\left(d_{\mathrm{Au}}q_{\mathrm{Au}}\right)\sin\left(d_{{\mathrm{SiO}}_{2}}q_{{\mathrm{SiO}}_{2}}\right)J_{\sigma }\\ & & +i\beta_{\mathrm{Au}}^{2}\cos^{2}\left(d_{\mathrm{Au}}q_{\mathrm{Au}}\right)\sin\left(d_{\mathrm{Au}}q_{\mathrm{Au}}\right)K_{\sigma }\\ & & +2\beta_{\mathrm{Au}}^{3}\beta_{{\mathrm{SiO}}_{2}}\cos^{3}\left(d_{\mathrm{Au}}q_{\mathrm{Au}}\right)L_{\sigma }, \end{array}$$

where the subindex σ should be replaced by N or D using

(8) $$\begin{array}{ccc} G_{N} & = & \beta_{\mathrm{Ce}-\mathrm{YIG}}^{2}+\left(\beta_{\mathrm{Si}}-\delta \right)\left(\beta_{\mathrm{air}}+\delta \right), \end{array}$$

(9) $$\begin{array}{ccc} G_{D} & = & \beta_{\mathrm{Si}}+\beta_{\mathrm{air}}, \end{array}$$

(10) $$\begin{array}{ccc} H_{N} & = & \beta_{\mathrm{Ce}-\mathrm{YIG}}^{2}\beta_{\mathrm{air}}+\beta_{\mathrm{Si}}\left(\beta_{\mathrm{Au}}^{2}+\beta_{\mathrm{air}}\delta \right)-\delta \left(\beta_{\mathrm{Au}}^{2}+\beta_{\mathrm{air}}\delta \right), \end{array}$$

(11) $$\begin{array}{ccc} H_{D} & = & \beta_{\mathrm{Au}}^{2}+\beta_{\mathrm{Si}}\beta_{\mathrm{air}}, \end{array}$$

(12) $$\begin{array}{ccc} J_{N} & = & \beta_{\mathrm{Au}}\sin\left(2d_{\mathrm{Au}}q_{\mathrm{Au}}\right)\{2i\beta_{{\mathrm{SiO}}_{2}}[\beta_{\mathrm{Ce}-\mathrm{YIG}}^{2}(\beta_{\mathrm{Au}}^{2}\\ & & +2\beta_{{\mathrm{SiO}}_{2}}^{2})\beta_{\mathrm{air}}+\beta_{\mathrm{Si}}(2\beta_{\mathrm{Au}}^{4}+2\beta_{{\mathrm{SiO}}_{2}}^{2}\beta_{\mathrm{air}}\delta \\ & & +\beta_{\mathrm{Au}}^{2}\left(\beta_{{\mathrm{SiO}}_{2}}^{2}+\beta_{\mathrm{air}}\delta \right))\\ & & -\delta (2\beta_{\mathrm{Au}}^{4}+2\beta_{{\mathrm{SiO}}_{2}}^{2}\beta_{\mathrm{air}}\delta +\beta_{\mathrm{Au}}^{2}\left(\beta_{{\mathrm{SiO}}_{2}}^{2}+\beta_{\mathrm{air}}\delta \right))]\\ & & \times \cos\left(d_{{\mathrm{SiO}}_{2}}q_{{\mathrm{SiO}}_{2}}\right)+\left(\beta_{\mathrm{Au}}^{4}+\beta_{\mathrm{Au}}^{2}\beta_{{\mathrm{SiO}}_{2}}^{2}+\beta_{{\mathrm{SiO}}_{2}}^{4}\right)\\ & & \times \left(\beta_{\mathrm{Ce}-\mathrm{YIG}}^{2}+\left(\beta_{\mathrm{Si}}-\delta \right)\left(\beta_{\mathrm{air}}+\delta \right)\right)\sin\left(d_{{\mathrm{SiO}}_{2}}q_{{\mathrm{SiO}}_{2}}\right)\}\\ & & +2\sin^{2}\left(d_{\mathrm{Au}}q_{\mathrm{Au}}\right)\{\beta_{\mathrm{Au}}^{2}\beta_{{\mathrm{SiO}}_{2}}\left(\beta_{\mathrm{Au}}^{2}+\beta_{{\mathrm{SiO}}_{2}}^{2}\right)(\beta_{\mathrm{Ce}-\mathrm{YIG}}^{2}\\ & & +\left(\beta_{\mathrm{Si}}-\delta \right)\left(\beta_{\mathrm{air}}+\delta )\right)\times \cos\left(d_{{\mathrm{SiO}}_{2}}q_{{\mathrm{SiO}}_{2}}\right)\\ & & -i[\beta_{\mathrm{Ce}-\mathrm{YIG}}^{2}\beta_{{\mathrm{SiO}}_{2}}^{4}\beta_{\mathrm{air}}+\beta_{\mathrm{Si}}\left(\beta_{\mathrm{Au}}^{6}+\beta_{{\mathrm{SiO}}_{2}}^{4}\beta_{\mathrm{air}}\delta \right)\\ & & -\delta \left(\beta_{\mathrm{Au}}^{6}+\beta_{{\mathrm{SiO}}_{2}}^{4}\beta_{\mathrm{air}}\delta \right)]\sin\left(d_{{\mathrm{SiO}}_{2}}q_{{\mathrm{SiO}}_{2}}\right)\}, \end{array}$$

(13) $$\begin{array}{ccc} J_{D} & = & \beta_{\mathrm{Au}}\sin\left(2d_{\mathrm{Au}}q_{\mathrm{Au}}\right)[2i\beta_{{\mathrm{SiO}}_{2}}(2\beta_{\mathrm{Au}}^{4}+2\beta_{\mathrm{Si}}\beta_{{\mathrm{SiO}}_{2}}^{2}\beta_{\mathrm{air}}\\ & & +\beta_{\mathrm{Au}}^{2}\left(\beta_{{\mathrm{SiO}}_{2}}^{2}+\beta_{\mathrm{Si}}\beta_{\mathrm{air}}\right))\cos\left(d_{{\mathrm{SiO}}_{2}}q_{{\mathrm{SiO}}_{2}}\right)\\ & & +\left(\beta_{\mathrm{Au}}^{4}+\beta_{\mathrm{Au}}^{2}\beta_{{\mathrm{SiO}}_{2}}^{2}+\beta_{{\mathrm{SiO}}_{2}}^{4}\right)\left(\beta_{\mathrm{Si}}+\beta_{\mathrm{air}}\right)\\ & & \times \sin\left(2d_{\mathrm{Au}}q_{\mathrm{Au}})\right]+2\sin^{2}\left(d_{\mathrm{Au}}q_{\mathrm{Au}}\right)[\beta_{\mathrm{Au}}^{2}\beta_{{\mathrm{SiO}}_{2}}\\ & & \times \left(\beta_{\mathrm{Au}}^{2}+\beta_{{\mathrm{SiO}}_{2}}^{2}\right)\left(\beta_{\mathrm{Si}}+\beta_{\mathrm{air}}\right)\cos\left(d_{{\mathrm{SiO}}_{2}}q_{{\mathrm{SiO}}_{2}}\right)\\ & & -i\left(\beta_{\mathrm{Au}}^{6}+\beta_{\mathrm{Si}}\beta_{{\mathrm{SiO}}_{2}}^{4}\beta_{\mathrm{air}}\right)\sin\left(d_{{\mathrm{SiO}}_{2}}q_{{\mathrm{SiO}}_{2}}\right)], \end{array}$$

(14) $$\begin{array}{ccc} K_{N} & = & [\left(\beta_{{\mathrm{SiO}}_{2}}^{2}-\beta_{\mathrm{Au}}^{2}\right)(\beta_{\mathrm{Ce}-\mathrm{YIG}}^{2}\beta_{\mathrm{air}}+\delta \left(\beta_{{\mathrm{SiO}}_{2}}^{2}-\beta_{\mathrm{air}}\delta \right)\\ & & +\beta_{\mathrm{Si}}\left(-\beta_{{\mathrm{SiO}}_{2}}^{2}+\beta_{\mathrm{air}}\delta \right))]+\{\beta_{\mathrm{Ce}-\mathrm{YIG}}^{2}\left(\beta_{\mathrm{Au}}^{2}+5\beta_{{\mathrm{SiO}}_{2}}^{2}\right)\beta_{\mathrm{air}}\\ & & +\beta_{\mathrm{Si}}\left[\beta_{{\mathrm{SiO}}_{2}}^{4}+5\beta_{{\mathrm{SiO}}_{2}}^{2}\beta_{\mathrm{air}}\delta +\beta_{\mathrm{Au}}^{2}\left(5\beta_{{\mathrm{SiO}}_{2}}^{2}+\beta_{\mathrm{air}}\delta \right)\right]\\ & & -\delta [\beta_{{\mathrm{SiO}}_{2}}^{4}+5\beta_{{\mathrm{SiO}}_{2}}^{2}\beta_{\mathrm{air}}\delta +\beta_{\mathrm{Au}}^{2}\left(5\beta_{{\mathrm{SiO}}_{2}}^{2}+\beta_{\mathrm{air}}\delta \right)]\}\\ & & \times \cos\left(2d_{{\mathrm{SiO}}_{2}}q_{{\mathrm{SiO}}_{2}}\right)-3i\beta_{{\mathrm{SiO}}_{2}}\left(\beta_{\mathrm{Au}}^{2}+\beta_{{\mathrm{SiO}}_{2}}^{2}\right)\\ & & \times \left[\beta_{\mathrm{Ce}-\mathrm{YIG}}^{2}+\left(\beta_{\mathrm{Si}}-\delta \right)\left(\beta_{\mathrm{air}}+\delta \right)\right]\sin\left(2d_{{\mathrm{SiO}}_{2}}q_{{\mathrm{SiO}}_{2}}\right), \end{array}$$

(15) $$\begin{array}{ccc} K_{D} & = & \left(\beta_{\mathrm{Au}}^{2}-\beta_{{\mathrm{SiO}}_{2}}^{2}\right)\left(\beta_{{\mathrm{SiO}}_{2}}^{2}-\beta_{\mathrm{Si}}\beta_{\mathrm{air}}\right)\\ & & +[\beta_{{\mathrm{SiO}}_{2}}^{4}+5\beta_{\mathrm{Si}}\beta_{{\mathrm{SiO}}_{2}}^{2}\beta_{\mathrm{air}}+\beta_{\mathrm{Au}}^{2}\left(5\beta_{{\mathrm{SiO}}_{2}}^{2}+\beta_{\mathrm{Si}}\beta_{\mathrm{air}}\right)]\\ & & \times \cos\left(2d_{{\mathrm{SiO}}_{2}}q_{{\mathrm{SiO}}_{2}}\right)-3i\beta_{{\mathrm{SiO}}_{2}}\left(\beta_{\mathrm{Au}}^{2}+\beta_{{\mathrm{SiO}}_{2}}^{2}\right)\\ & & \times \left(\beta_{\mathrm{Si}}+\beta_{\mathrm{air}}\right)\sin\left(2d_{{\mathrm{SiO}}_{2}}q_{{\mathrm{SiO}}_{2}}\right), \end{array}$$

(16) $$\begin{array}{ccc} L_{N} & = & \beta_{{\mathrm{SiO}}_{2}}\left[\beta_{\mathrm{Ce}-\mathrm{YIG}}^{2}+\left(\beta_{\mathrm{Si}}-\delta \right)\left(\beta_{\mathrm{air}}+\delta \right)\right]\\ & & \times \cos\left(2d_{{\mathrm{SiO}}_{2}}q_{{\mathrm{SiO}}_{2}}\right)-i[\beta_{\mathrm{Ce}-\mathrm{YIG}}^{2}\beta_{\mathrm{air}}+\beta_{\mathrm{Si}}(\beta_{{\mathrm{SiO}}_{2}}^{2}\\ & & +\beta_{\mathrm{air}}\delta )-\delta \left(\beta_{{\mathrm{SiO}}_{2}}^{2}+\beta_{\mathrm{air}}\delta \right)]\sin\left(2d_{{\mathrm{SiO}}_{2}}q_{{\mathrm{SiO}}_{2}}\right) \end{array}$$

(17) $$\begin{array}{ccc} L_{D} & = & \beta_{{\mathrm{SiO}}_{2}}\left(\beta_{\mathrm{Si}}+\beta_{\mathrm{air}}\right)\cos\left(2d_{{\mathrm{SiO}}_{2}}q_{{\mathrm{SiO}}_{2}}\right)\\ & & -i\left(\beta_{{\mathrm{SiO}}_{2}}^{2}+\beta_{\mathrm{Si}}\beta_{\mathrm{air}}\right)\sin\left(2d_{{\mathrm{SiO}}_{2}}q_{{\mathrm{SiO}}_{2}}\right) \end{array}$$

and

(18) $$\begin{array}{ccc} \beta_{i} & = & \eta_{i}q_{i}, \end{array}$$

(19) $$\begin{array}{ccc} q_{i} & = & \sqrt{\frac{\omega^{2}}{c^{2}}\frac{1}{\eta_{i}}-k_{x}^{2}}, \end{array}$$

(20) $$\begin{array}{ccc} \eta_{\mathrm{Si}} & = & \frac{1}{\varepsilon_{\mathrm{Si}}}, \end{array}$$

(21) $$\begin{array}{ccc} \eta_{\mathrm{Ce}-\mathrm{YIG}} & = & \frac{\varepsilon_{\mathrm{mo}}}{\varepsilon_{\mathrm{mo}}^{2}-g^{2}}, \end{array}$$

(22) $$\begin{array}{ccc} \eta_{{\mathrm{SiO}}_{2}} & = & \frac{1}{\varepsilon_{{\mathrm{SiO}}_{2}}}, \end{array}$$

(23) $$\begin{array}{ccc} \eta_{\mathrm{Au}} & = & \frac{1}{\varepsilon_{\mathrm{Au}}}, \end{array}$$

(24) $$\begin{array}{ccc} \delta & = & \frac{imgk_{x}}{\varepsilon_{\mathrm{mo}}^{2}-g^{2}}. \end{array}$$

This set of equations, in particular Equation (6), indicate that we can actively manipulate the matching-condition through changes in $q_{\mathrm{Ce}-\mathrm{YIG}}$. Since we are interested in the TMOKE, these changes are achieved by flipping M (through the use of an external magnetic field), i.e., by sign changes of *m* in Equation (24).

**Figure 4:**
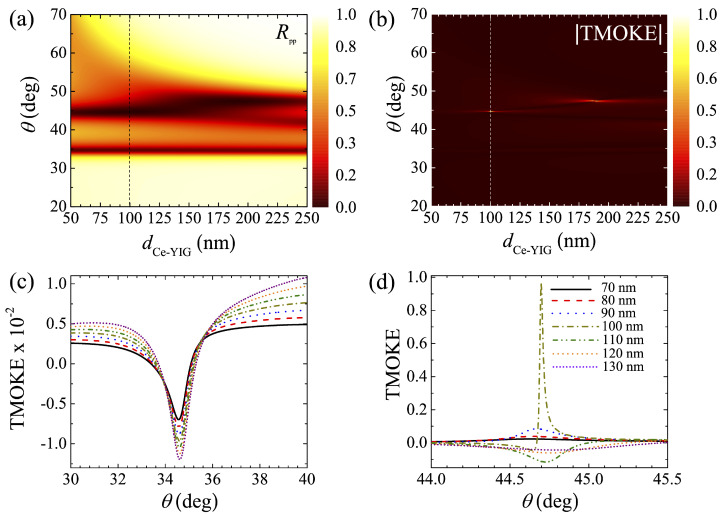
(**a**) Reflectance $R_{\mathrm{pp}}$ for m=0 (i.e., the demagnetized system) and (**b**) $\left|\mathrm{TMOKE}\right|$ as functions of the incident angle (θ) and the MO layer thickness ($d_{\mathrm{Ce}-\mathrm{YIG}}$). TMOKE associated with the (**c**) BPP${}_{0}$ ($34.71^{\circ }$) and (**d**) BPP${}_{1}$ ($44.69^{\circ }$) resonances for different $d_{\mathrm{Ce}-\mathrm{YIG}}$ values. All the calculations in this figure were performed using $d_{m}=20.42$ nm and $d_{\mathrm{Ce}-\mathrm{YIG}}=100$ nm.

The finite element method (FEM), using the commercial software COMSOL Multiphysics, and the SMM, were used for full electromagnetic simulations of the system in Figure 3. the numerical results are presented for the TMOKE and R(±M), with an inset showing the differences in R(+M) and R(−M) due to changes in the ATR-SPR matching-condition (induced by MO activity). In these calculations, we replace the first slab from Figure 2, made of SiO${}_{2}$, by a MO slab of Ce−YIG with thickness $d_{\mathrm{Ce}-\mathrm{YIG}}=100$ nm. The TMOKE for this system associated to the resonance BPP${}_{1}$ has an amplitude of 0.977, as seen from the vertical scale on the right-hand side in Figure 3. In contrast, the TMOKE associated with BPP${}_{0}$ exhibits negligible behavior (see Figure 4c), which can be understood in two different, yet complementary, ways: (i) numerically, the higher reflectance value associated with BPP${}_{0}$ produces a very low TMOKE amplitude, as numerically studied in [17]; (ii) physically, the near-zero reflectance associated with BPP${}_{1}$ indicates a near-perfect incident light-HMM coupling, which, in turn, enhances the MO activity of the nanostructure [17]. We can observe a higher near-field amplitude inside the MO slab when comparing the field profiles shown in the lower panel of Figure 3.

**Figure 5:**
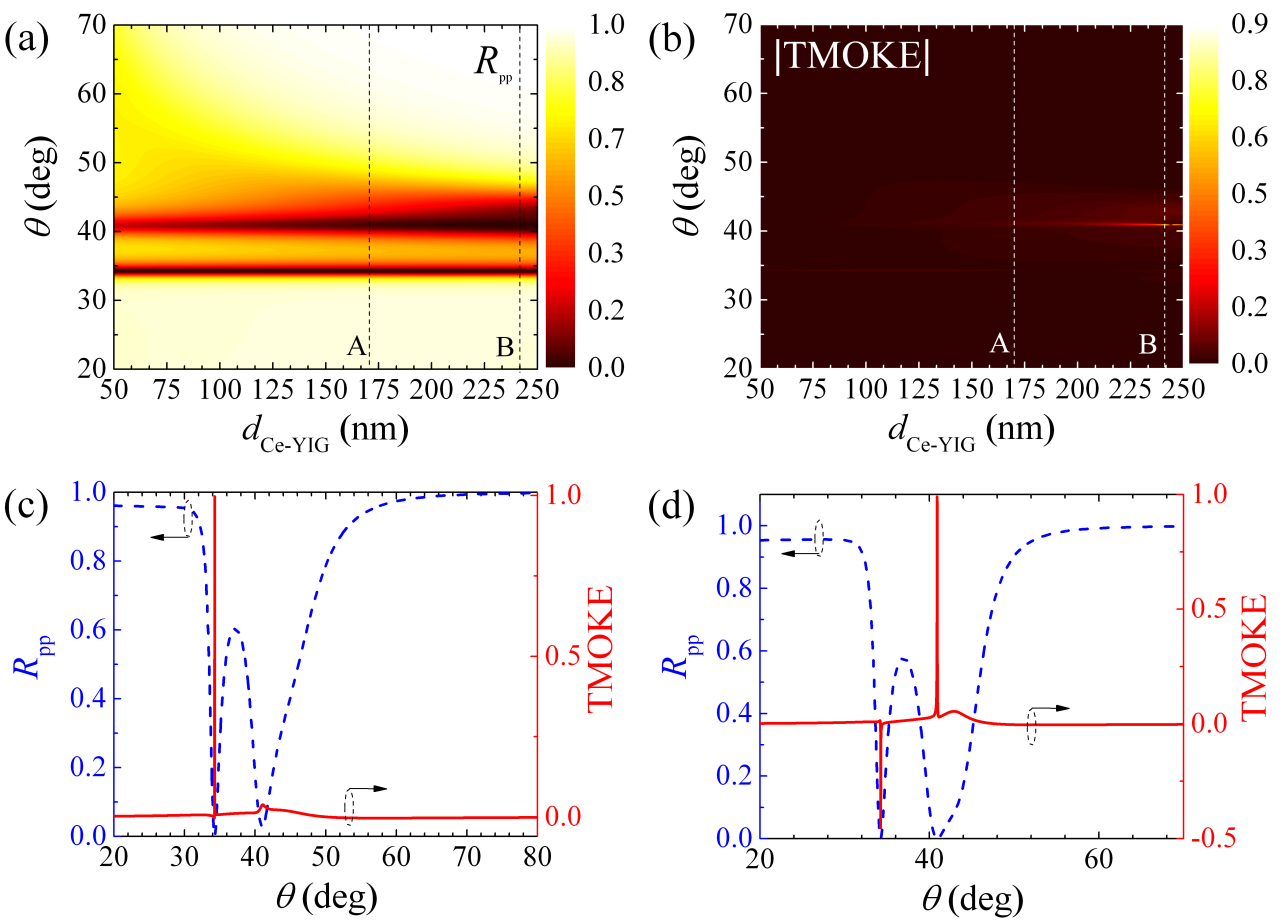
(**a**) Reflectance $R_{\mathrm{pp}}$ for m=0 (i.e., the demagnetized system) and (**b**) $\left|\mathrm{TMOKE}\right|$ as functions of the incident angle (θ) and the MO layer thickness ($d_{\mathrm{Ce}-\mathrm{YIG}}$) for the system with $d_{m}=28$ nm and $d_{d}=50$ nm. The vertical dotted lines (labeled **A** and **B**) in (**a**,**b**) denote results for $d_{\mathrm{Ce}-\mathrm{YIG}}=170$ nm and $d_{\mathrm{Ce}-\mathrm{YIG}}=242$ nm, which are plotted in (**c**,**d**), respectively.

**Figure 6:**
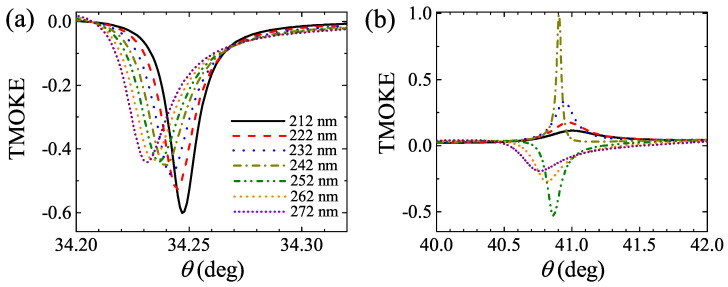
Numerical analysis of TMOKE associated with the (**a**) BPP${}_{0}$ and (**b**) BPP${}_{1}$ resonances from the vertical dotted line **B** in Figure 5.

As shown in Figure 4a,b, we calculated $R_{\mathrm{pp}}\left(m=0\right)$ and $\left|\mathrm{TMOKE}\right|$ as a function of the incident angle (θ) and $d_{\mathrm{Ce}-\mathrm{YIG}}$ for a thorough analysis of BPP${}_{0}$ and BPP${}_{1}$. These results (calculated for $d_{m}=20.42$ nm) indicate that the resonant angle of BPP${}_{0}$ is insensitive to $d_{\mathrm{Ce}-\mathrm{YIG}}$, as corroborated in Figure 4c, whilst BPP${}_{1}$ suffers large changes with relatively small variations (∼10 nm) of $d_{\mathrm{Ce}-\mathrm{YIG}}$, as displayed in Figure 4d. Nevertheless, the negligible TMOKE amplitudes of BPP${}_{0}$ in Figure 4c are not interesting for applications. Therefore, we also carried out a numerical sweep of $d_{m}$ using the SMM. In particular, Figure 5a,b show $R_{\mathrm{pp}}\left(m=0\right)$ and $\left|\mathrm{TMOKE}\right|$ as a function of θ and $d_{\mathrm{Ce}-\mathrm{YIG}}$ for $d_{m}=28$ nm. Once again, it is seen that the resonance angle for BPP${}_{0}$ is almost insensitive to $d_{\mathrm{Ce}-\mathrm{YIG}}$. Moreover, the BPP${}_{1}$ is less affected by $d_{\mathrm{Ce}-\mathrm{YIG}}$ in this case, in contrast to the results in Figure 4. Interestingly, we found that there are some $d_{m}$ values where BPP${}_{0}$ and BPP${}_{1}$ simultaneously become near-zero, with two near-maximum TMOKE amplitudes, as presented in Figure 5c,d for $d_{\mathrm{Ce}-\mathrm{YIG}}=170$ nm and $d_{\mathrm{Ce}-\mathrm{YIG}}=242$ nm, respectively. It is worth noting that the sharper TMOKE resonances, when compared with the reflectance spectra, could find application in future MO materials for sensing/metrology devices. The quality factor *Q*, defined as the ratio between the central resonance (i.e., θ of the resonance peak) and the full-width at half-maximum (FWHM), was obtained as Q=6386.26 for the TMOKE peak in Figure 5c. The *Q* values for the TMOKE peaks in Figure 5d were obtained as 1606.58 and 959.2 for the BPP${}_{0}$ and BPP${}_{1}$ resonances, respectively. Despite these advantages, we note that our concept is sensitive to manufacturing errors. Figure 6a,b show the TMOKE associated with the structure in Figure 5 for $d_{\mathrm{Ce}-\mathrm{YIG}}$ varying from 212 nm to 272 nm, where changes are visualized for both BPP${}_{0}$ and BPP${}_{1}$ resonances.

## 4. Conclusions

In summary, we have demonstrated that BPP modes in non-magnetic type II HMMs can be used for giant enhancement of the MO activity of an adjacent dielectric MO slab. In particular, we considered a dielectric MO spacer between the prism coupler and the non-magnetic multilayer HMM. Rigorous analytical calculations were used to show that the physical principle of this mechanism stems from the magnetic manipulation of the ATR-SPR matching-condition for BPP excitation. Moreover, using full electromagnetic simulations, through the software COMSOL Multiphysics and SMM, we also showed that the TMOKE values can be maximized by optimizing the ATR-SPR matching-condition. This last optimization was achieved by tuning the MO layer thickness, which affects the phase of the evanescent ATR-SPR wave coupling. It is significant that our concept uses a single Si-compatible dielectric MO spacer, between the Si substrate and the multilayer HMM, enabling a new means of manufacturing CMOS-integrable hyperbolic MO metamaterials for future active nanophotonic applications.

## Data Availability

Not applicable.
